# Follicular thyroid carcinoma invades venous rather than lymphatic vessels

**DOI:** 10.1186/1746-1596-5-8

**Published:** 2010-01-22

**Authors:** Xiaoqi Lin, Bing Zhu, Yulin Liu, Jan F Silverman

**Affiliations:** 1 Department of Pathology, Northwestern Memorial Hospital, Northwestern University, Chicago, USA; 2 Department of Pathology, Allegheny General Hospital, Pittsburgh, USA

## Abstract

Follicular thyroid carcinoma (FTC) tends to metastasize to remote organs rather than local lymph nodes. Separation of FTC from follicular thyroid adenoma (FTA) relies on detection of vascular and/or capsular invasion. We investigated which vascular markers, CD31, CD34 and D2-40 (lymphatic vessel marker), can best evaluate vascular invasion and why FTC tends to metastasize via blood stream to remote organs. Thirty two FTCs and 34 FTAs were retrieved for evaluation. The average age of patients with FTA was 8 years younger than FTC (p = 0.02). The female to male ratio for follicular neoplasm was 25:8. The average size of FTC was larger than FTA (p = 0.003). Fourteen of 32 (44%) FTCs showed venous invasion and none showed lymphatic invasion, with positive CD31 and CD34 staining and negative D2-40 staining of the involved vessels. The average number of involved vessels was 0.88 ± 1.29 with a range from 0 to 5, and the average diameter of involved vessels was 0.068 ± 0.027 mm. None of the 34 FTAs showed vascular invasion. CD31 staining demonstrated more specific staining of vascular endothelial cells than CD34, with less background staining. We recommended using CD31 rather than CD34 and/or D2-40 in confirming/excluding vascular invasion in difficult cases. All identified FTCs with vascular invasions showed involvement of venous channels, rather than lymphatic spaces, suggesting that FTCs prefer to metastasize via veins to distant organs, instead of lymphatic vessels to local lymph nodes, which correlates with previous clinical observations.

## Introduction

Follicular thyroid carcinoma (FTC) accounts for 10 - 17% of clinically evident thyroid malignancies [1-4]. It is more common in women, and tends to occur in patients in the fifth decade[1]. Survival is better in women and in patients younger than 40 years for male and 50 years for female [4-6]. Separation of FTC from follicular thyroid adenoma (FTA) is based on detection of vascular and/or capsular invasion[1]. The vascular invasion is almost never evident grossly[7]. Microscopically, the vessels should be located in or immediately outside the capsule (rather than within the tumor), and contain one or more clusters of tumor cells attached to the wall with protrusion into the lumen[1,7]. Often, the intravascular tumor foci are covered by endothelium, in a fashion similar to that of an ordinary thrombus[7]. The endothelial markers, such as CD31, factor VIII-related antigen, and Ulex europaeus, have been used in identifying vascular invasion [8-10]. When vascular invasion is identified in FTCs, there is a prognostic significance based on the number of vessels involved (< 4 or ≥ 4 vascular invasion)[7,11-15].

Clinically, FTC tends to spread via blood stream, especially to the bones and lungs, and rarely to regional lymph nodes[1,16-20]. The skeletal metastases are usually multicentric but have a predilection for the shoulder girdle, sternum, skull, and iliac bone[21,22]. These metastases are common in the FTCs demonstrating extensive vascular invasion, but occur in fewer than 5% FTCs with minimal vascular invasion, and develop in less than 1% of the tumors diagnosed as carcinoma only on the basis of minimal capsular invasion[14,23,24]. Thirteen percentage of FTC smaller than 3 cm, 19% FTC between 3 to 6 cm, and 33% FTC > 6 cm show vascular invasion[25]. Up to 10 % of patients with follicular or Hurthle cell carcinoma have tumors that aggressively invade structures in the neck or produce distant metastasis[26]. The metastases may exhibit a better differentiated appearance than the primary tumor, to the point of simulating normal thyroid as an expression of terminal differentiation (so-called "metastasizing adenoma", "malignant adenoma", or "metastasizing goiter")[7]. The majority, however, have poorly differentiated features, at least at the architectural level[20].

Occasionally, it can be challenging to detect vascular invasion on hematoxylin and eosin (H&E) stained slides. Although vascular immunohistochemical (IHC) markers such as CD31[27,28], Factor VIII[9,10], Ulex europaeus[8] and CD34[27,29] have been used to identify vascular invasion in malignant neoplasms, the diagnostic value of these vascular markers compared to a specific lymphatic IHC marker, D2-40, in FTC has not been investigated [30,31]. In this study we investigated which vascular markers, CD31, CD34 and D2-40, can best identify vascular invasion in FTC, and studied whether venous or lymphatic vessels were involved. To date, no study has demonstrated a predilection of FTC for invading venous versus lymphatic vessels.

## Materials and methods

### Selection of Cases

The institutional review board of Allegheny General Hospital, Pittsburgh, PA approved the study. Thirty four follicular thyroid adenomas (FTA) and 32 follicular thyroid carcinomas (FTC) from 2000 to 2008 were retrieved from the hospital computer system. All FTCs were diagnosed when vascular and/or capsular invasion was histologically present. All patients were followed-up from 1 to 8 years, and none of the patients showed distal metastasis.

The average tumor size of FTA and FTC was 2.8 ± 0.9 cm with a range from 1.1 to 5.5 cm and 4.4 ± 2.0 with a range from 1.4 to 7.5 cm, respectively (P = 0.003). The average age of patients with FTA was approximately 8 years younger than those with FTC (48.2 ± 11.3 vs. 56.7 ± 11.6 years old, P = 0.02). The average age for male and female patients with FTC was 55.2 ± 9.75 and 56.2 ± 13.0, respectively (P = 0.91). The female to male ratio with follicular thyroid neoplasm was 25:8, suggesting female predominance. The female to male ratios of FTA and FTC were 15:2 and 5:3, respectively (p = 0.000007).

### Pathologic evaluation and immunohistochemistry

Formalin-fixed (10 % buffered formalin), routinely processed, hematoxylin and eosin-stained (H&E) tissue sections including the entire capsule of the tumor were evaluated independently by 2 pathologists (XL and YL). Two blocks with capsule vascular invasion identified on H&E stained slides or suspicious for vascular invasion from each case were chosen for immunohistochemical study.

Paraffin-embedded blocks were sectioned, deparaffinized, rehydrated, and blocked with methanolic 3% hydrogen peroxide. Antigen retrieval was performed in citrate buffer (pH 6.0). The immunohistochemical stains (IHC) for CD31 (catalog number CMA338, clone jc70a, Mouse IgG1, no dilution, Cell Marquee, Hot Springs, AR), CD34 (catalog number 790-2927, clone QBEnd/10, mouse IgG1, no dilution, Ventana, Tucson, AR), and D2-40 (catalog number 730-01, clone D2-40, mouse IgG1, 1:50 dilution, Signet, Dedham, MA) on these slides were performed in an automated immunostainer with appropriate positive and negative controls. The detection was performed with Iview DAB detection kit (Catalog number 760-091, Vantana, Tucson, AZ). All slides were counterstained with hematoxylin and then were evaluated independently by 2 pathologists (XL and YL).

### Statistics

The vascular invasion results from reviewing H&E and IHC stained slides by two pathologists were compared. Chi-square test and Student T test were used for statistical analysis.

## Results

### Identification of vascular invasion of follicular thyroid neoplasms

**Figure 1:**
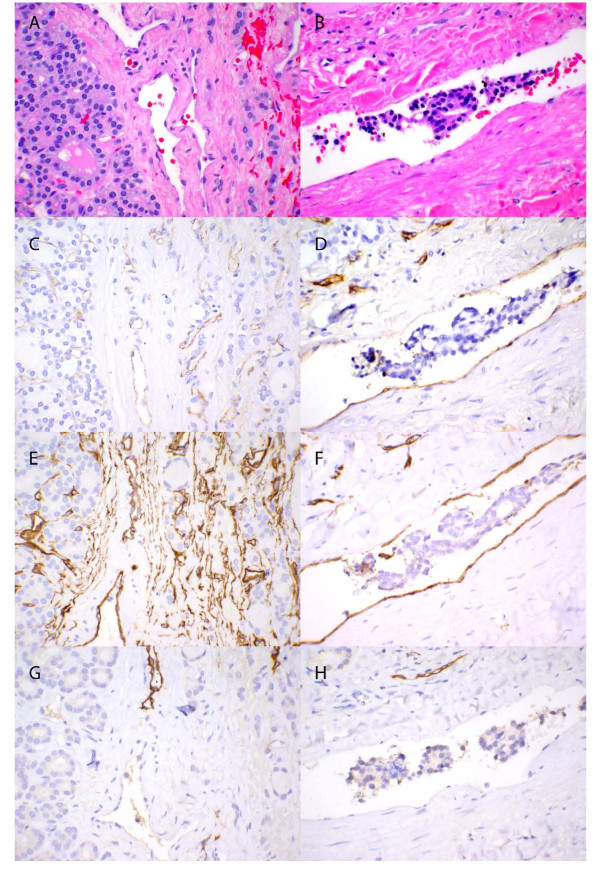
**Follicular thyroid adenoma (A, C, E and G) and follicular thyroid carcinoma (B, D, F and H) (400×)**. Hematoxylin and eosin stains (A and B), CD31 stains (C and D), CD34 stains (E and F), and D2-40 stains (G and H).

In this study, we identified vascular invasion in 13 FTC cases and no FTA cases by H&E stained slides (Table 1 and Figure 1). IHC stain with CD31 and CD34 identified one more case in the FTC group, but no additional case in the FTA group. In the FTC group, one case was reported as "vascular invasion is equivocal", but capsular invasion was present. Reexamination of the equivocal case confirmed vascular invasion on repeat H&E slides, which further was confirmed with IHC stains for CD31 and CD34. These results suggested that IHC stain with CD31 and CD34 could help identify more vascular invasion and identify vascular invasion more accurately, and that IHC staining with CD31 or CD34 should be done especially if vascular invasion was equivocal. We also found that CD34 stained many non-endothelial cells, which occasionally compromised evaluation of vascular invasion. In contrast, CD31 was more specific than CD34 for vascular endothelial cells, making evaluation of vascular invasion much clearer.

**Table 1:** IHC identification of vascular invasion of follicular thyroid neoplasms

Neoplasms	**No**.	H&E	D2-40	CD31	CD34
Follicular Carcinoma	32	41 % (13/32)*	0 % (0/32)	44 % (14/32)*	44 % (14/32)*
Follicular Adenoma	34	0 % (0/34)	0 % (0/34)	0 % (0/34)	0 % (0/34)

*: P < 0.01. Chi-square test.

The average number of vessels demonstrating vascular invasion was 0.88 ± 1.29 with a range from 0 to 5. The average diameter of involved vessels was 0.068 ± 0.027 mm.

### The vessels invaded by FTC were venous rather than lymphatic

Antibodies against CD31[27,28] and CD34[27,29] are two widely-used markers of vascular endothelial cells and stain endothelial cells of both blood and lymphatic vessels, in contrast to D2-40 which stains only the endothelial cells of lymphatic vessels[30,31]. In this study, we found that all the vessels invaded by the FTCs were stained with CD31 and CD34 and none were stained with D2-40, supporting that venous rather than lymphatic invasion was occurring (Table 1 and Figure 1). These results indicated FTC prefers to invade the venous rather than the lymphatic vessels.

## Discussion

The diagnosis of FTC is contingent upon identifying capsular and/or vascular invasion[1]. Identification of the number of vessels involved by FTC is also important to predict prognosis (< 4 or ≥ 4 vascular invasion)[7,11]. In this study, we found that antibody against CD31 is better than antibodies against CD34 and D2-40 in identifying FTC vascular invasion and that FTC prefers to invade the venous rather than the lymphatic vessels.

Occasionally, it can be difficult to identify vascular invasion on H&E stained slides[1,7]. One of our cases showed capsular invasion, but was initially equivocal for vascular invasion. However, vascular invasion was confirmed with IHC stains for CD31 and CD34. In addition, IHC stains for CD31 and CD34 identified vascular invasion in one case that was missed in the H&E stained slides. Therefore, any suspicion for vascular invasion should be confirmed by IHC stains for CD31 and CD34, since IHC stains for CD31 and CD34 can more accurately and readily identify vascular invasion. We found that CD31 is preferable to CD34 for detecting vascular invasion of FTC due to less background staining. It is important to identify the vascular invasion, as the degree of vascular invasion by FTC has important prognostic implications[7,11].

Clinically, FTC is observed to metastasize hematogenously to distant organs in 7 - 40% of patients, with special preference to the lung and bones, rather than via lymphatics to regional nodes[2,19,20,25,32-34]. To date, no study has shown a preference of invading venous blood vessels versus lymphatic vessels by FTC. Antibodies against CD31 and CD34 stain both venous and lymphatic endothelial cells [27-29]. However, an antibody against D2-40 is a very sensitive and specific marker for lymphatic endothelial cells[30,31]. In this study, the endothelial cells of the vascular invasions in FTC were positive CD31 and CD34 and negative for D2-40, strongly suggesting that vascular invasion of FTC involves venous but not lymphatic vessels, which correlates with previous clinical observations[19,20]. However, the reason for this phenomenon is unknown, since papillary carcinoma is known to have a strong proclivity to involve lymphatic vessels with regional lymph node metastasis. In this study, we found that both D2-40 negative venous and D2-40 positive lymphatic vessels are present in the FTC and FTA capsules, excluding the possibility of lack of lymphatic vessels in the capsules. Future study on the preference of venous invasion by the FTC is important in order to further clarify the clinical behavior of FTC and to predict prognosis of FTC. In this study, no case showed distal and local metastasis during 1 to 8 years follow-up. Two cases with 4 or 5 venous vessels involved respectively also did not show distant metastasis 6 years after excision of FTC, but our observation may be limited by the relatively short follow-up. Another reason that may contribute to our finding is that with incoming use of thyroid fine needle biopsy to evaluate thyroid nodule in recent years, more FTC cases are potentially being diagnosed and treated at an earlier stage.

It was reported that FTC is more common in women, and tends to occur in patients in the fifth decade[1]. In this study, we had similar findings that the female to male ratio with follicular neoplasm was 25:8, with in 15:2 female to male ratio in FTA and 5:3 in FTC. These results not only suggested that follicular neoplasm especially FTA is much more common in female than male patients, but also male patients have higher risk than female patients to have FTC once they have thyroid follicular neoplasm.

In this study, we found that the average age of patients with FTA was about 8 years younger than those with FTC (48.2 ± 11.3 vs. 56.7 ± 11.6 years old, P = 0.02, which was statistical significance). The average age of FTC patients is similar to a previous published report[1].

Four or more vessels involved by FTC has been proposed to have a worse prognosis[7,11-15]. Dr. Ghossein also has suggested to avoid the use of the term "minimally invasive" for those aggressive encapsulated follicular neoplasms with extensive angioinvasion, because the FTC with extensive vascular invasion implies a higher risk for recurrence[12]. It was suggested that the number of foci of vascular invasion should be mentioned in the pathology reports on FTCs, with a note regarding the increased risk of recurrence if at least 4 foci of vascular invasion are observed[12]. In our study, we had 2 cases with 4 and 5 vessels involved, respectively. Neither of the two patients show metastasis or local recurrence at 6 years after lobectomy and subtotal thyroidectomy. Tumor size greater than 4 cm has been shown to have worse prognosis[12]. In our study, we found that FTCs were on average significantly larger than FTA (p = 0.003).

In summary, we found that FTC invades venous vessels in the capsule rather than lymphatic vessels, which correlates with clinical observation that FTC more frequently metastasize via the blood stream to distant organs rather than regional lymph nodes. We recommended using IHC stain for CD31 rather than CD34 and D2-40 to confirm vascular invasion in difficult cases and predict distant metastasis and prognosis.

## Competing interests

The authors declare that they have no competing interests.

## Authors' contributions

Dr. X Lin participated in its design, carried out the study, analyzed the data and drafted the manuscript. Dr. Y Lin participated in its design and analyzed the data. Drs. B Zhu and JF Silverman drafted the manuscript. All authors read and approved the final manuscript.
